# High−risk lineages shape the resistome and virulome of multidrug−resistant *Pseudomonas aeruginosa*

**DOI:** 10.3389/fcimb.2026.1843668

**Published:** 2026-06-17

**Authors:** Kristyna Novakova, Iva Sukkar, Jana Palkovicova, Katerina Fiserova, Iva Vagnerova, Vendula Pudova, Milan Kolar

**Affiliations:** 1 Department of Microbiology, University Hospital Olomouc, Olomouc, Czechia; 2 Department of Microbiology, Faculty of Medicine and Dentistry, Palacky University Olomouc, Olomouc, Czechia; 3 Central European Institute of Technology, University of Veterinary Sciences Brno, Brno, Czechia; 4 Department of Microbiology, Faculty of Medicine, Charles University, Pilsen, Czechia

**Keywords:** multidrug-resistant, *Pseudomonas aeruginosa*, ST175, ST233, ST235, ST357

## Abstract

Multidrug−resistant (MDR) and extensively drug−resistant (XDR) *Pseudomonas aeruginosa* strains remain a major threat in clinical settings, yet no contemporary genomic data are available from the Olomouc region, Czech Republic. We performed whole−genome sequencing of 157 MDR isolates obtained from 133 patients hospitalized at two tertiary−care hospitals in Olomouc between April 2020 and April 2024. The aim of this study was to delineate the clonal structure, resistance and virulence repertoires shaping the local epidemiology. Of the 157 isolates, 81 (51.6%) met XDR criteria, most commonly exhibiting susceptibility to colistin (37/81, 45.7%). Four dominant high−risk sequence types (STs) were identified: ST175 (n = 53), ST357 (n = 49), ST233 (n = 25), and ST235 (n = 11). ST357 comprised two phylogenetically and functionally distinct sublineages: a major cluster (43/49) carrying *bla*_IMP−7_ and *bla*_OXA−2_, and a smaller *bla*_VIM−2_−positive cluster (5/49) lacking *bla*_OXA−2_, *oprD*, *soxR*, and the *pvc* operon, correlating with an XDR phenotype and reduced biofilm−associated virulence potential. All ST357 isolates carried the cytotoxic *exoU* gene. Both *bla*_IMP−7_ and *bla*_VIM−2_ were chromosomally encoded. ST175 isolates lacked acquired carbapenemase genes, except for a single *bla*_VIM−2_−positive isolate. This lineage carried the highest number of virulence genes, dominated by adherence− and biofilm−associated determinants together with the characteristic *exoS*^+^/*exoT*^+^/*exoY*^+^ type III secretion system profile. All ST233 isolates carried *bla*_VIM−2_ and exhibited an XDR phenotype, consistent with globally disseminated VIM−2 lineages. ST235 displayed the greatest diversity of carbapenemase genes, including *bla*_GES−14_ (5/11), *bla*_GES−29_ (1/11), and *bla*_VIM−1_ (1/11). This represented the first identification of a *bla*_VIM−1_−positive ST235 isolate in Olomouc University Hospital, and, to our knowledge, the first documented occurrence of a VIM−1−producing ST235 strain in the Czech Republic. All ST235 isolates were *exoU*−positive. This study provides a recent whole−genome analysis of MDR/XDR *P. aeruginosa* in the Czech Republic, integrating resistome, virulome and molecular epidemiology. Our findings demonstrate that the local population is shaped by multiple high−risk lineages with distinct resistance-virulence profiles, underscoring their clinical relevance and the necessity of genomic surveillance to guide therapy and prevent hospital transmission.

## 1 Introduction

Multidrug-resistant (MDR) *Pseudomonas aeruginosa* (PSAE) represents one of the most significant etiological agents associated with hospital-acquired infections. The management of these infections frequently necessitates the use of reserve antibiotics, a practice that inevitably contributes to the rising prevalence of MDR strains (Almeida et al., 2024). Between 2020 and 2024, the global epidemiological landscape of MDR PSAE was further shaped by increased antibiotic pressure in intensive care units (ICUs), partly driven by the COVID-19 pandemic. Analyses at the ICU level documented increased antibiotic utilization during pandemic waves, along with changes in class-specific use. These pressures are relevant for selection and propagation of MDR PSAE and even more dangerous strains, extensively drug-resistant (XDR) PSAE in critical care settings (Çaşkurlu et al., 2024; Wójkowska-Mach et al., 2024; Tsuzuki et al., 2025). The clinical impact of MDR and XDR isolates is substantial, as these strains are directly associated with adverse patient outcomes in the same high-risk environments. They are associated with high mortality rates in ventilator-associated pneumonia and bloodstream infections, imposing a significant burden on healthcare systems. Recent cohort data highlight the challenging treatment of MDR and XDR PSAE, including reliance on novel beta-lactam/beta-lactamase inhibitor combinations (Horcajada et al., 2019; Kothari et al., 2023; Mendes Pedro et al., 2024). Nosocomial strains of PSAE harbour primary and secondary resistance mechanisms, which pose a significant obstacle to existing therapeutic approaches. The wide array of resistance mechanisms in PSAE is largely attributable to the genomic plasticity of these organisms. This genomic variability drives the rapid evolutionary dynamics of *Pseudomonas* spp., thereby continuously compromising the development and clinical utility of novel antimicrobial agents through the emergence of newly induced resistance mechanisms (Pachori et al., 2019; Karampatakis et al., 2025; Choudhury and Andam, 2026).

Molecular epidemiology and phenotypic characterisation of MDR PSAE have been documented in hospital settings worldwide to shed light on life-threatening lineages and to prevent their spread (Magalhães et al., 2020; Strateva et al., 2024). In Central Europe, sequence type (ST) 357 has emerged as a clinically relevant lineage. Czech ST357 isolates producing IMP−7 have been described, and clonal spread within hospital settings has been observed (Papagiannitsis et al., 2017). Recent genomic analyses further identified ST357 among colistin−resistant isolates, underscoring its classification as a high−risk clone. Although only one isolate out of twenty was identified as ST357, this finding remains clinically significant (Talat et al., 2024). Globally, ST235 remains one of the most prevalent high−risk clones strongly associated with carbapenem resistance mediated by *bla*_VIM_ and *bla*_IMP_. ST357 and ST235 are characterised by the presence of virulence gene, *exoU*, which confers cytotoxicity and is linked to severe clinical outcomes (Treepong et al., 2018; Horcajada et al., 2019; Fischer et al., 2020; de Sales et al., 2025). ST175, although less widespread than ST235, is an important lineage in Spain and other European regions, typically characterised by *bla*_VIM_ variants and by the presence of exotoxin genes *exoS* and *exoT*. These exotoxins are associated with invasiveness and immune evasion of strains (Cholley et al., 2011; Oliver et al., 2015; Roy Chowdhury et al., 2017; Recio et al., 2020; Doumith et al., 2022; Sendra et al., 2024). Finally, ST233 has gained importance through regional and international dissemination. It is frequently linked to VIM−2 production and has been associated with emerging XDR phenotypes, underscoring its growing clinical relevance (Aguilar-Rodea et al., 2022; Doumith et al., 2022). These STs illustrate how resistance determinants (*bla*_VIM_, *bla*_IMP_, *bla*_NDM−1_) and virulence genes (*exoU*, *exoS*, *exoT*, *exoY*) contribute to drive persistence, dissemination, and clinical severity in hospital−acquired infections (Swain et al., 2025). Beyond carbapenemase and exotoxin production, resistance and persistence in hospital environments in high-risk clones is reinforced by efflux pumps, porin mutations (notably OprD), and biofilm formation. These mechanisms interact with quorum sensing and virulence regulation, complicating eradication and promoting relapses. This co-existence of resistance determinants, exotoxins ExoU, ExoS, ExoT and ExoY and virulence factors participating in biofilm formation explains why these clones are classified as “high-risk” and why their eradication from hospital environments remains particularly challenging (Elfadadny et al., 2024; Zhang et al., 2025).

The high genomic plasticity of PSAE limits the discriminatory power of standard methods. Whole−genome sequencing (WGS) provides higher resolution than routine approaches such as PCR and enables comprehensive analysis of resistance genes, virulence factors and emerging new extended-spectrum beta-lactamases (ESBL) and metallo-beta-lactamases (MBL) variants (Magalhães et al., 2020; Cortes-Lara et al., 2021; Strateva et al., 2024). The study focuses on the characterisation of MDR PSAE circulating in Czech hospitals, with an emphasis on the high−risk lineages ST357, ST175, ST233, and ST235.

## 2 Materials and methods

This retrospective study analysed 157 MDR PSAE isolates collected between April 2020 to April 2024. The isolates were obtained from patients hospitalised primarily in the Intensive Care Department of the Military Hospital Olomouc (MHO) and the Department of Anaesthesiology, Resuscitation and Intensive Care (DARIC) of the University Hospital Olomouc (UHO). The isolates were obtained from various types of patient-derived samples collected across multiple hospital departments (**Supplementary Table 1**; **Supplementary Table 2**). For isolates originating from the same patient, only those collected at least 90 days after the previous sampling, or those exhibiting a different phenotypic susceptibility profile, were included. Isolates obtained from the same type of specimen from the same patient that shared an identical phenotypic profile were excluded from the analysis.

The final percentage prevalence of each diagnosis was calculated based on the number of isolates. As multiple isolates could be obtained from a single patient and associated with different diagnoses, the diagnostic percentages do not correspond to the number of patients.

### 2.1 Antimicrobial susceptibility testing and storage of isolates

All isolates were cultured on Mueller–Hinton agar (TRIOS, Czech Republic) prior to testing. Identification of all isolates was performed by MALDI-TOF MS using the Microflex LT/SH instrument and Biotyper software (Bruker, Germany), following the manufacturers’ instructions. Routine susceptibility testing to eight antibacterial agents (piperacillin/tazobactam [PPT], ceftazidime [CTZ], cefepime [CPM], meropenem [MER], tobramycin [TOB], amikacin [AMI], colistin [COL], ciprofloxacin [CIP] was carried out using the standard microtiter broth dilution method according to EUCAST criteria (European Committee on Antimicrobial Susceptibility Testing, [[NoYear]]). The E-test (Bio-Rad, Hercules, CA, USA) was employed to determine susceptibility to ceftazidime/avibactam [CZA], and ceftolozane/tazobactam [CNT] in accordance with the manufacturer’s guidelines. All isolates were screened using the NG−TEST CARBA−5 assay (NG Biotech, France) to determine the presence of any carbapenemases included in the test panel.

Based on the susceptibility profiles of PSAE isolates to clinically relevant antimicrobial agents, isolates meeting the MDR criteria were selected and stored at −80 °C in ITEST tubes containing a commercially available cryogenic medium (ITEST-plus, Czech Republic).

### 2.2 Genetic analysis of MDR PSAE isolates

#### 2.2.1 Short-read sequencing and data analysis

Genomic DNA was extracted using the NucleoSpin Tissue kit (Macherey-Nagel, Germany). The extracted DNA was sent to the Institute of Applied Biotechnologies (IAB, Czech Republic) for library preparation and subsequent WGS. DNA libraries were prepared using TruSeq DNA PCR-Free kit (Illumina, Inc., USA) (Illumina TruSeq DNA PCR‑Free Reference Guide, 2017) and sequenced on the Illumina NovaSeq X Plus with 4,5M PE reads/sample (2×150 bp paired-end sequencing).

Raw reads were quality trimmed using Trimmomatic v0.39 (Bolger et al., 2014) and *de novo* assembled with SPAdes v3.12.0 (Bankevich et al., 2012). The assembled data were screened by ABRicate v1.0.1 for antimicrobial resistance genes using ResFinder database (2025-10-23) (Zankari et al., 2012) and the Comprehensive Antibiotic Resistance Database (CARD, 2025-05-29) (Alcock et al., 2023). Plasmid content was assessed using the PlasmidFinder database (2025-04-09) (Carattoli et al., 2014), and virulence genes were identified using the VFDB (2025-07-25) (Chen et al., 2005). For all databases, the minimum thresholds were set to 90% nucleotide identity and coverage of reference sequences. Gene annotation and examination of the genetic surroundings of the target genes were performed using Geneious software v9.1.8 (Biomatters, Auckland, New Zealand) (Kearse et al., 2012).

Given the clinical relevance of the OprD porin in mediating carbapenem susceptibility, this gene was analysed separately, as it is not included in ResFinder or CARD database. To determine the presence and integrity of *oprD*, the custom database was created with the reference *oprD* sequence obtained from the *Pseudomonas* Genome Database (ID 104678; available at https://pseudomonas.com/) and all isolates were screened using ABRicate v1.0.1. The hits were considered valid if both nucleotide identity and coverage exceeded 90%.

#### 2.2.2 MLST and phylogenetic analysis

STs of isolates were assigned using the MLST tool (2025-02-06) (Larsen et al., 2012) with the PSAE-specific database (Jolley et al., 2018). Phylogenetic analysis was performed by RAxML v8.2.13 tool (Stamatakis, 2014) using Prokka-annotated v1.14.6 (Seemann, 2014), multi-aligned by PIRATE v1.0.5 (Bayliss et al., 2019). The GTR-GAMMA model of heterogeneity was used, identified as the best−fit model by jModelTest v2.1.10 (Darriba et al., 2012). The robustness of the resulting tree topology was evaluated using 500 bootstrap replicates. The final phylogenetic tree was visualized using iTOL v7 (Letunic and Bork, 2024).

Based on pairwise SNP distances, isolates were categorised into genomic groups. Thresholds were adjusted to reflect the high genomic plasticity of PSAE and the diversity observed across the full dataset. SNP thresholds were selected based on published evidence on within-host evolution in PSAE, where longitudinal sampling consistently demonstrates the accumulation of tens of SNP (Feliziani et al., 2014; Marvig et al., 2015). A threshold of ≤40 SNP was applied to define SNP-based epidemiological categories, representing isolate pairs compatible with recent transmission. This value was selected based on the distribution of SNP distances observed in our dataset, which showed substantial genomic variability within the ST357 background. Similar data-driven approaches have been used in previous genomic epidemiology studies, where SNP cut-offs are defined according to the characteristics of the analysed dataset rather than applied universally (Stimson et al., 2019). A secondary threshold of ≤60 SNP was used to identify within-host evolutionary clusters, but only when isolates originated from the same patient. This threshold was selected based on the distribution of SNP distances in our dataset and is consistent with the levels of diversity observed in longitudinal studies of PSAE (Feliziani et al., 2014; Bianconi et al., 2019). Broader sublineages were defined as groups of isolates separated by 40–200 SNP, representing deeper evolutionary structure. Isolates separated by >200 SNP from all others were classified as divergent lineages.

The sublineage characterisation heatmap was generated using the Flourish data visualization platform, Heatmap v7.4.4 (Canva UK Operations Ltd, United Kingdom) (Canva UK Operations Ltd., [[NoYear]]) based on gene presence/absence and functional category scores. Scores represent the proportion of genes detected in each sublineage relative to the total number of detected genes in each functional category in this analysis (score 0.0–1.0).

#### 2.2.3 Long-read sequencing and data analysis

Five representative isolates of the most prevalent STs and different carbapenemase producers were selected for long-read sequencing to fully reconstruct surroundings of genes encoding carbapenemases. DNA was extracted using NucleoBond HMW DNA kit (Macherey-Nagel). Subsequently, DNA library was prepared using SQK-RBK114 rapid barcoding kit (Oxford Nanopore Technologies (ONT), Ltd., Oxford, England) following manufacturer’s protocol, loaded onto a FLO-MIN114 flow cell (ONT) and sequenced on MinION Mk1B platform (ONT).

The obtained raw reads were demultiplexed and adaptor-trimmed using Porechop v0.2.4 (Wick et al., 2017), and quality-trimmed (Q ≤ 9) by BBduk from BBMap package v39.01 (https://sourceforge.net/projects/bbmap/). *De novo* assembly was performed with Unicycler v0.4.8 (Vaser et al., 2017) in hybrid mode using both long and short reads (Vaser et al., 2017). Obtained assembled sequences were polished by Racon v1.4.20 (Vaser et al., 2017) and medaka v1.2.3 (Lee et al., 2021) with long reads and by Pilon v1.23 (Walker et al., 2014) using short reads.

The positions of carbapenamase and other resistance genes in representative isolates were assessed using ABRicate with database ResFinder. The completely assembled sequences were automatically annotated using Bakta v1.11.4 (Schwengers et al., 2021) and the genetic surroundings of antimicrobial resistance genes (ARGs) of interest were inspected and corrected in Geneious Prime 2025.2.2 (Kearse et al., 2012) based on the comparison with ISfinder (Siguier et al., 2006), Conserved Domain Database (Lu et al., 2020), TnCentral (Ross et al., 2021) and NCBI database (O’Leary et al., 2024) using BLAST algorithms (BLASTN, BLASTX, BLASTP). Annotated sequences were compared based on their nucleotide sequences and visualised using clinker (Gilchrist and Chooi, 2021).

Sequences of previously published (Papagiannitsis et al., 2017) carbapenemase-producing PSAE (CP056774, KY860566, KY860570 and KY860573) were downloaded from GenBank database to assess the conservation of the carbapenemase-associated genetic context.

## 3 Results

### 3.1 Clinical characteristics of 157 PSAE isolates

All 157 PSAE isolates were obtained from 133 patients hospitalised in MHO and UHO. Most patients originated from the 15th Ward, Department of Anaesthesiology, Resuscitation and Intensive Care Medicine (DARIC-15) department of UHO (21.1%, 28/133) and the Post−acute Intensive Care Unit (PA-ICU) of MHO (17.3%, 23/133). The remaining patients were distributed across other departments (**Supplementary Table 2**). PSAE isolates obtained from urine (31.9%, 50/157) and lower respiratory tract samples (30.6%, 48/157) were the most common specimen types.

Among the 157 isolates, 61.1% (96/157) were associated with active infection, while 38.9% (61/157) represented colonization. Lower respiratory tract infections were the most frequent clinical manifestation (33.3%, 32/96), followed by urinary tract infections (25.0%, 24/96). Sepsis occurred in 37.5% (36/96) of infection episodes. Colonization most commonly involved urinary catheters (45.9%, 28/61) and the lower respiratory tract (36.1%, 22/61).

PSAE infection or colonization was associated with COVID-19 in 20.3% (27/133) of patients. Among these, 37.0% (10/27) represented colonization and 55.6% (15/27) active infection. From two patients, two PSAE isolates were recovered from each, with one isolate classified as the etiological agent and the other as a colonizer. The predominant infection was hospital−acquired pneumonia (HAP) developing after COVID-19 pneumonia (40.7%, 11/27).

### 3.2 Antibiotic resistance in 157 PSAE isolates

All 157 isolates met the criteria for multidrug resistance and were resistant to meropenem (100.0%, 157/157). The highest resistance rates were observed for ceftazidime (98.1%, 154/157), ciprofloxacin (97.5%, 153/157), cefepime (96.8%, 152/157), tobramycin (93.0%, 146/157), and piperacillin/tazobactam (87.9%, 138/157). In contrast, susceptibility to colistin (98.1%, 154/157) and amikacin (65.0%, 102/157) was largely retained.

XDR phenotypes were identified in 51.6% (81/157) of isolates. Among XDR isolates, susceptibility was most often retained to colistin alone (45.7%, 37/81) or to colistin combined with amikacin (30.9%, 25/81). Two isolates (1.3%, 2/157) were pan-resistant.

### 3.3 Genetic analysis of MDR PSAE isolates

#### 3.3.1 Resistance determinants

In all isolates classified as VIM− or IMP−positive by CARBA−5, the presence of the corresponding genes was confirmed. Five different carbapenemase genes were detected in the PSAE collection. The most common detected MBL genes were *bla*_IMP−7_ and *bla*_VIM-2_, while the lowest detection frequency showed *bla*_GES-29_ and *bla*_VIM-1_ genes, each detected in one isolate (**Table 1**). Among the AmpC−type beta−lactamases, the most frequently detected variant was *bla*_PDC−374_ (93.6%, 147/157). Among the OXA−type beta−lactamases, *bla*_OXA−1018_ was the most common (33.8%, 53/157), followed by *bla*_OXA−846_ (31.2%, 49/157; **Supplementary Table 3**).

**Table 1:** Overview of phenotypic resistance profiles and associated resistance gene repertoires of isolates carrying *bla*_IMP−7_ and variants of *bla*_GES_ and *bla*_VIM_ genes.

Carbapenemase genes	Prevalence	Phenotypic resistance profile	Resistance gene profile
*bla*_GES-14_	3.2% (5/157)	PPT, CTZ, CPM (3/5), MER, TOB, AMI (3/5), CIP, CZA (2/5), CNT	*aac(6’)-Ib9, bla*_OXA-488_*, bla*_PDC-374_*, crpP* (4/5)
*bla*_GES-29_	0.6% (1/157)	CTZ, CPM, MER, TOB, AMI, CIP, CNT	*aac(6’)-Ib9, bla*_OXA-488_ *, bla*_PDC-374_ *, crpP*
*bla*_IMP-7_	27.4% (43/157)	PPT (26/43), CTZ, CPM, MER, TOB, AMI (15/43), CIP, CZA, CNT	*aac(6’)-Ib* (10/43), *aac(6’)-Ib7* (3/43), *aac(6’)-Ib9* (2/43), *bla*_OXA-2_, *bla*_OXA-846_, *bla*_PDC-374_, *crpP* (42/43)
*bla*_VIM-1_	0.6% (1/157)	PPT, CTZ, CPM, MER, TOB, CZA, CNT	*aac(6’)-Ib7, bla*_OXA-488_ *, bla*_PDC-374_
*bla*_VIM-2_	22.9% (36/157)	PPT, CTZ, CPM, MER, TOB, AMI (30/36), CIP, CZA, CNT (35/36)	*aac(3)-Id* (25/36), *aac(6’)-Ib7* (5/36), *aac(6’)-Il* (25/36), *ant(2’’)-Ia* (8/36), *aph(3’)-VIa* (2/36), *bla*_LCR-1_ (6/36), *bla*_OXA-486_ (25/36), *bla*_PDC-374_ (31/36), *bla*_PDC-55_ (5/36), *crpP* (31/36)

Antibiotic abbreviations indicate resistance phenotype observed within each group. Values in parentheses denote the number of resistant isolates when resistance was not uniform across the group.

Given the high rates of phenotypic resistance to tobramycin 93% (146/157) and amikacin 35% (55/157), two acquired aminoglycoside resistance determinants were particularly relevant. Tobramycin-specific gene *ant(2’’)−Ia* was detected in 38.2% (60/157) of isolates, with only a single *ant(2’’)−Ia*–positive isolate remaining susceptible to tobramycin, indicating strong genotype–phenotype correlation. The N-acetyltransferase *aac(6’)−II* was detected in 18.5% (29/157) of isolates, all of which were resistant to tobramycin and nearly all to amikacin, except for two isolates. The chromosomally encoded gene *aph(3’’)−IIb* was identified in all isolates (**Supplementary Table 3**).

The *oprD* gene was not detected in 4.5% isolates (7/157). Most of these (71.4%, 5/7) belonged to the VIM−2–positive ST357 subgroup. Remaining two isolates were assigned to ST463 (14.3%, 1/7) or newly assigned ST6927 (14.3%, 1/7; **Supplementary Table 4**).

#### 3.3.2 Phylogenetic analyses of all 157 isolates corresponding to their ST

Across the entire dataset, the phylogenetic tree (Supplementary data **Supplementary Figure 1**) resolved several well−defined clonal lineages corresponding to the dominant STs, with ST175 and ST357 forming the largest and most cohesive clusters. ST233 and ST235 each formed deeply separated monophyletic groups, reflecting long−standing evolutionary divergence from the major high−prevalence lineages. The smaller ST654 group clustered tightly together and shared a branch with a single ST463 isolate, suggesting a related sublineage. In contrast, the unassigned isolate occupied a long, isolated branch with large SNP distances to all major clusters, indicating a sporadic, genetically distinct lineage with no evidence of recent shared ancestry. The phylogenetic tree contained several dominant clonal clusters together with multiple rare and highly divergent lineages.

#### 3.3.3 Characterisation of sequence types

Among the PSAE collection, 15 distinct STs were observed. The most common sequence types were ST175 (33.8%, 53/157), ST357 (31.2%, 49/157), ST233 (15.9%, 25/157), and ST235 (7.0%, 11/157). ST357 was the predominant lineage when assessed per patient (34.6%, 46/133).

Isolates assigned to ST175, ST235, ST357 and ST654 were more frequently identified as etiological agents, reaching 66.0% (35/53), 72.7% (8/11), 63.3% (31/49) and 60.0% (3/5), respectively. These lineages were also associated with higher sepsis rates. In contrast, ST233 was mainly associated with colonisation. Although these numerical differences suggested potential trends, the overall distribution of etiological versus colonising isolates across STs was not statistically significant (p = 0.13). Infection type varied across sequence types, with ST175 linked to respiratory infections and ST357 to urinary tract infections.

##### 3.3.3.1 Comprehensive genomic, phylogenetic and epidemiological analysis of ST357

**Figure 1:**
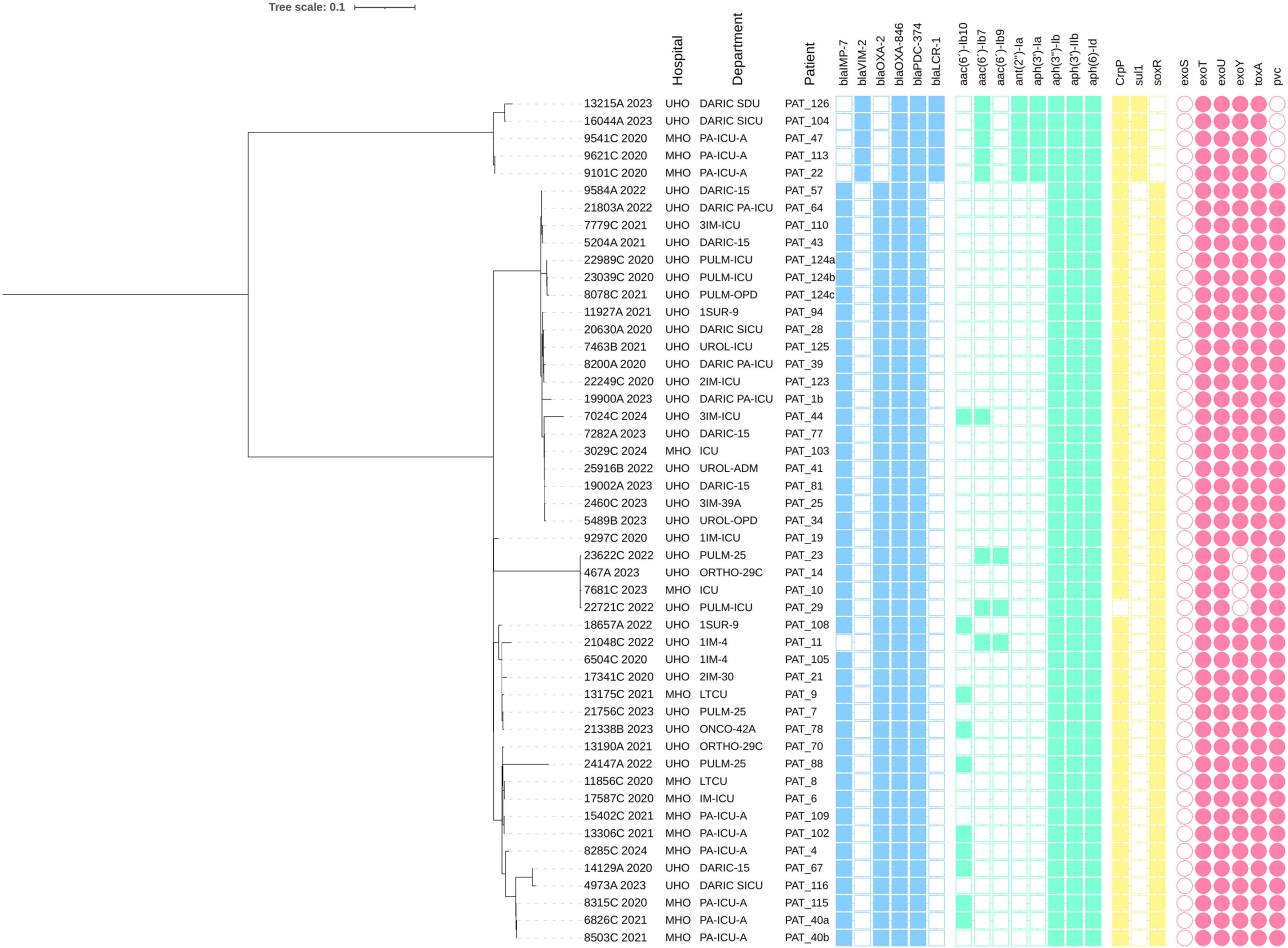
Phylogenetic relatedness of ST357 isolates. Columns show isolate identifier, hospital, department, patient identifier, and presence/absence of the respective genes. Department abbreviations are listed in **Supplementary Table 2**. Squares represent resistance determinants and circles represent virulence−associated genes. Blue colour indicates beta−lactamase genes, green aminoglycoside resistance genes, yellow other resistance determinants, and pink colour virulence factors.

The most prevalent ST357 lineage (based on the number of patients, 49 isolates obtained from 46 patients) comprised two major sublineages distinguished by the presence of MBL genes. A larger subgroup of isolates within this ST carried *bla*_IMP−7_ (43/49), whereas the remaining isolates encoded *bla*_VIM−2_ (5/49). One isolate lacked both *bla*_VIM−2_ and *bla*_IMP−7_. This isolate was the only one who showed additional susceptibility to CZA and CNT. Pairwise distances between the *bla*_VIM−2_ and *bla*_IMP−7_ ST357 isolates were extremely large (typically ~5,000–7,500 SNP), confirming that these groups represent unrelated evolutionary lineages despite sharing the same sequence type. The phylogenetic tree resolved two main branches: one composed exclusively of *bla*_VIM−2_ –positive isolates, and a second, larger branch comprising all *bla*_IMP−7_–positive isolates together with the single MBL−negative isolate. Apart from differences in MBL gene content, isolates belonging to this ST uniformly carried the exotoxin genes *exoT*, *exoU* and mostly *exoY* (**Figure 1**). Of these isolates, 71.4% (35/49) were obtained from patients hospitalised at UHO and 28.6% (14/49) at MHO.

All *bla*_IMP−7_–positive ST357 isolates (43/49) showed phenotypic resistance to CTZ, CPM, MER, TOB, CIP, CZA and CNT. Resistance to PPT, AMI and COL were observed in 60.5% (26/43), 34.9% (15/43) and 7% (3/43) of isolates, respectively. All isolates within this subgroup carried *bla*_OXA-2_, *bla*_OXA-846_ and *bla*_PDC-374_. On the contrary to *bla*_VIM-2_-positive isolates, the *bla*_IMP−7_ subgroup generally lacked additional aminoglycoside-modifying enzyme genes beyond *aph(3’’)-Ib*, *aph(3’)-IIb* and *aph(6)-Id*, with only a minority carrying *aac(6’)-Ib10*, *aac(6’)-Ib7* and *aac(6’)-Ib9*.

Phylogenetic analysis confirmed high genomic diversity among ST357 *bla*_IMP−7_–positive isolates. Pairwise SNP distances ranged from 8 to 56 SNP among isolates obtained from the same patient. Despite this range, all patient−related isolates formed a single monophyletic clade and shared a highly similar accessory genome profile, consistent with intra−host microevolution rather than unrelated lineages. The largest genomic cluster consisted of 11 isolates, represented a multi−patient, multi−ward group and notably included three isolates originating from a single patient (PAT_124), illustrating intra−host microevolution embedded within a broader transmission−compatible cluster. Two isolates from patient PAT_40 together with one isolate from a different patient (PAT_115), hospitalised at the same department and ward, suggested a genomic cluster that spans both intra−host diversity and potential inter−patient transmission. The subsequent cluster consisted of four isolates and was characterised by the absence of the virulence−associated gene *exoY*, indicating a distinct accessory genome signature within this group. SNP distances within this group ranged from 18-31. These isolates originated from different patients from multiple wards, which may indicate repeated transmission events or patient movement across hospital units.

The *bla*_VIM-2_-positive isolates (5/49) formed a distinct ST357 sublineage, with pairwise SNP distances ranging from 32 to 164 SNP. Although these values indicate close phylogenetic relatedness, they exceed ≤40 SNP to define putative epidemiological transmission clusters. On the contrary to isolates carrying *bla*_IMP-7_, all five *bla*_VIM-2_-positive isolates carried class D beta−lactamase gene *bla*_LCR−1_ but lacked *bla*_OXA-2_, *oprD* gene, *soxR* regulator gene associated with the MexGHI−OpmD efflux pump (Lister et al., 2009) and paerucumarin synthesis genes (*ptxR*, *pvcA–D*). Phenotypically, all five isolates were resistant to all tested antibiotics except colistin, which is consistent with their resistome profile and indicates that this cluster belongs to the XDR ST357 sublineage.

##### 3.3.3.2 Comprehensive genomic, phylogenetic and epidemiological analysis of ST175

**Figure 2:**
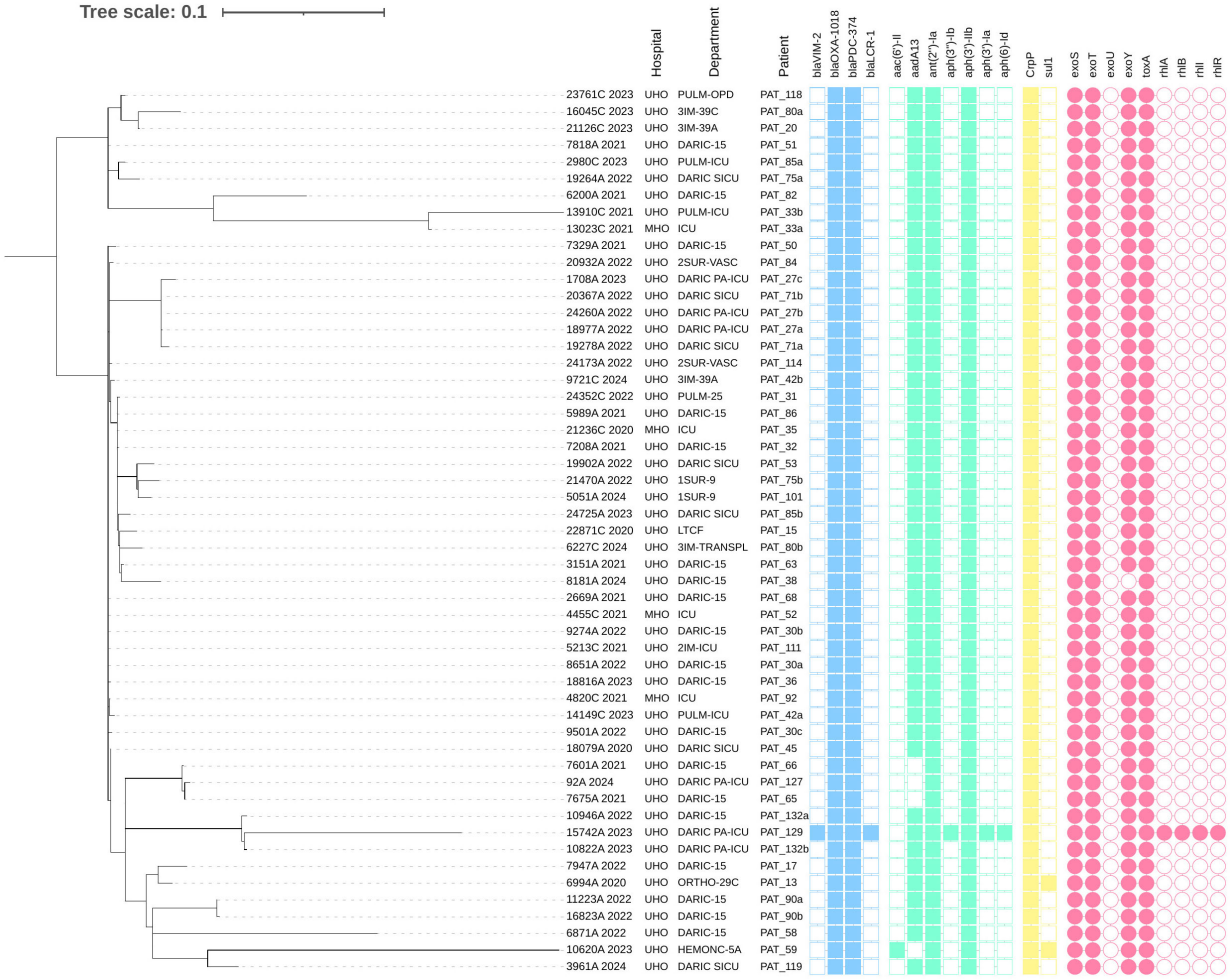
Phylogenetic relatedness of ST175 isolates. Columns show isolate identifier, hospital, department, patient identifier, and presence/absence of the respective genes. Department abbreviations are listed in **Supplementary Table 2**. Squares represent resistance determinants and circles represent virulence−associated genes. Blue colour indicates beta−lactamase genes, green aminoglycoside resistance genes, yellow other resistance determinants, and pink colour virulence factors.

When assessed by isolate counts, the most prevalent was ST175, composed of 53 isolates obtained from 41 patients. Most isolates (92.3%, 49/53) were obtained from patients hospitalised at UHO, with the remaining 7.7% (4/53) originating from MHO. All ST175 isolates, with the exception of a single isolate, carried only beta-lactamase genes *bla*_OXA−1018_ and *bla*_PDC−374_. The exceptional isolate harboured, in addition to *bla*_OXA−1018_ and *bla*_PDC−374_, the carbapenemase gene *bla*_VIM−2_ and the beta−lactamase C gene *bla*_LCR−1_. This isolate also possessed aminoglycoside resistance genes (*aph(3’’)−Ib*, *aph(3’)−Ia* and *aph(6)−Id*) that were not detected in the remaining ST175 isolates, and it was the only isolate in the dataset that remained susceptible only to colistin. All ST175 isolates carried the MGE−associated gene *ant(2’’)−Ia* and the chromosomally encoded gene *aph(3’)−IIb*. In addition, 92.5% (49/53) of isolates harboured *aadA13* gene. One isolate also carried the *aac(6’)−Il* gene. Analysis of virulence−associated genes showed that all ST175 isolates carried the exotoxin genes *exoS*, *exoT* and *exoY*, apart from a single isolate lacking *exoY*. Notably, the *bla*_VIM−2_−positive isolate uniquely harboured the rhamnolipid biosynthesis genes *rhlA* and *rhlB*, which were absent from all other ST175 isolates. This isolate also carried the quorum−sensing regulatory genes *rhlI* and *rhlR*, which were likewise not detected in the rest of the ST175 collection (**Figure 2**). All ST175 isolates carried a stop codon in *oprD* gene at position 142.

Across the full ST175 dataset, pairwise SNP distances ranged from 11 to 1566 SNP, indicating extremely high genomic diversity within this sequence type. When isolates differing only by ≤40 SNP were excluded, pairwise SNP distances among the remaining isolates ranged from 54 to 1566 SNP, with a median of 615 SNP, demonstrating that most non−cluster isolates belong to deeply divergent genomic lineages. In several patients, multiple isolates were recovered, including one case in which three isolates originated from a single individual. Their pairwise SNP distances frequently exceeded the intra−host threshold of 60 SNP defined in this study. This substantial divergence among isolates originating from the same host may indicate pronounced within−patient genomic heterogeneity and suggests the presence of multiple co−existing variants rather than a single clonal population.

##### 3.3.3.3 Characterisation of ST233

**Figure 3:**
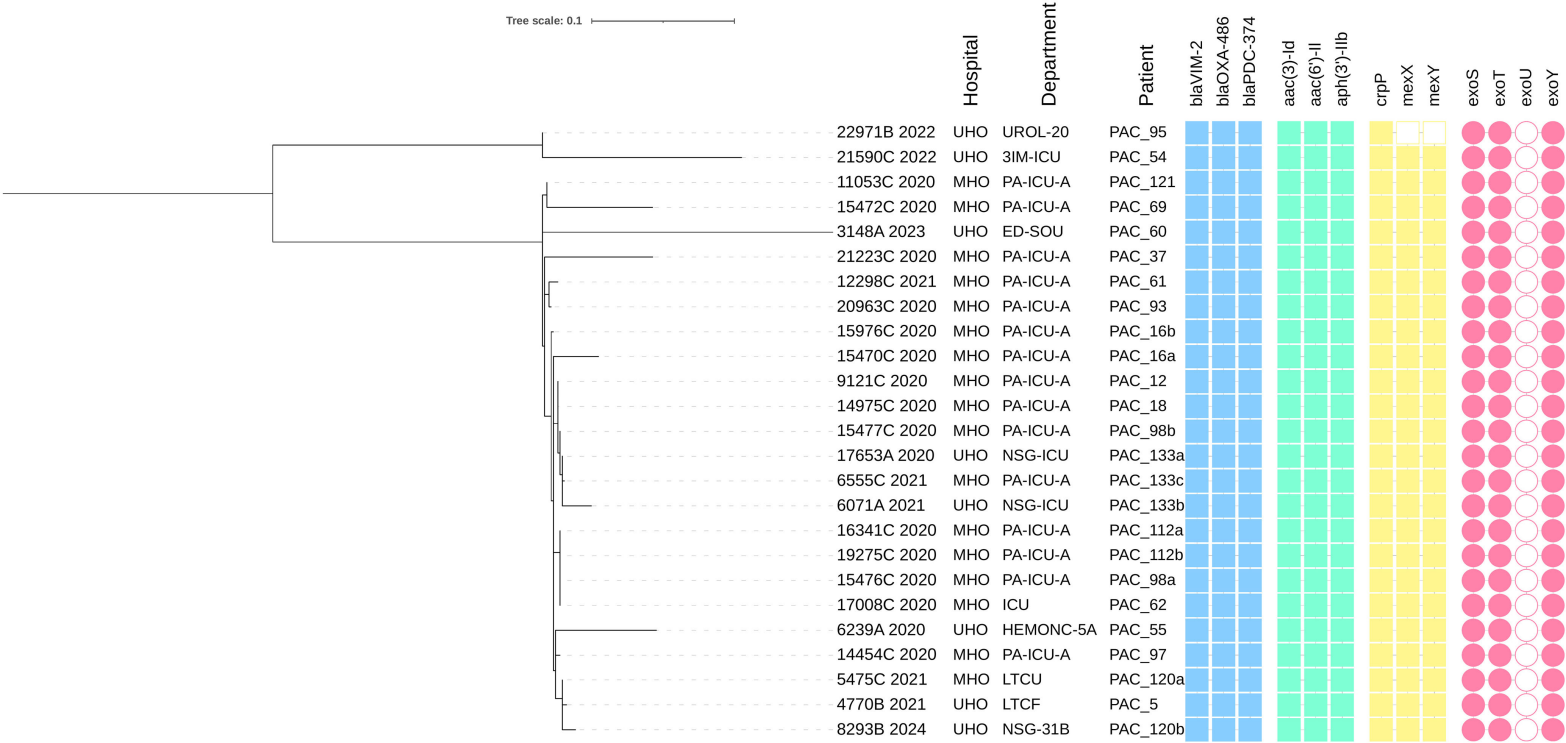
Phylogenetic relatedness of ST233 isolates. Columns show isolate identifier, hospital, department, patient identifier, and presence/absence of the respective genes. Department abbreviations are listed in **Supplementary Table 2**. Squares represent resistance determinants and circles represent virulence−associated genes. Blue colour indicates beta−lactamase genes, green aminoglycoside resistance genes, yellow other resistance determinants, and pink colour virulence factors.

The third major group in our dataset comprised ST233 isolates (25 isolates from 19 patients). All ST233 isolates were phenotypically susceptible exclusively to colistin classifying them as XDR. A single exception was one isolate, which exhibited an amikacin MIC of 16 mg/L, corresponding to the susceptibility breakpoint. This isolate was also the only one lacking the *mexX* and *mexY* efflux pump genes associated with aminoglycoside resistance. All 25 isolates harboured *bla*_VIM−2_, *bla*_OXA−486_ and *bla*_PDC−374_. Aminoglycoside resistance determinants detected across this ST included *aac(3)-Id, aac(6’)-Il* and the chromosomal *aph(3’)−IIb* gene. Isolates belonging to this ST were further characterised by the presence of the exotoxin genes *exoS*, *exoT* and *exoY* (**Figure 3**).

Pairwise SNP distances among ST233 isolates were low, with the most frequent value being 10 SNP. This supports the high genetic homogeneity observed within this sequence type. Nineteen isolates formed a tight cluster, with pairwise SNP distances consistently low and minimum SNP mostly ranging from one to five. This cluster was epidemiologically linked and predominantly associated with the PA−ICU−A ward, indicating sustained intra−unit transmission. The remaining ST233 isolates formed a genetically distinct sublineage, with substantially higher SNP distances (>45 SNP) and without direct epidemiological linkage. These isolates represented independent introductions rather than onward transmission.

##### 3.3.3.4 Characterisation of ST235

**Figure 4:**
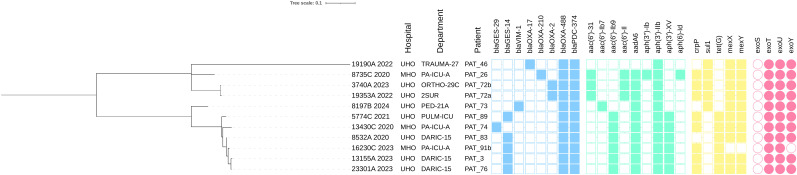
Phylogenetic relatedness of ST235 isolates. Columns show isolate identifier, hospital, department, patient identifier, and presence/absence of the respective genes. Department abbreviations are listed in **Supplementary Table 2**. Squares represent resistance determinants and circles represent virulence−associated genes. Blue colour indicates beta−lactamase genes, green aminoglycoside resistance genes, yellow other resistance determinants, and pink colour virulence factors.

ST235 isolates exhibited substantial genetic diversity, forming two distinct phylogenetic groups (**Figure 4**). The first phylogenetic branch comprised six isolates with pairwise SNP distances ranging from 10 to 77 SNP. Within this branch, a subset of four isolates showed low pairwise SNP distances (10–34 SNP), suggesting a potential clonal link. These isolates shared a conserved resistome including *aac(6’)−Ib9*, *aph(3’)−IIb*, *aph(3’)−XV*, *bla*_GES-14_, *bla*_OXA−488_, *bla*_PDC−374_ and *tet(G)*, consistent with their close phylogenetic relatedness. The only genetic difference identified was the absence of the efflux determinants *mexX* and *mexY*, together with the exotoxin gene *exoY*, in one isolate and the absence of *crpP* in another. These isolates originated from three patients hospitalised at DARIC−15 and one patient from PA−ICU−A, located in different hospitals. Patient transfers between units are common and may have facilitated inter−institutional transmission.

A second cluster comprised four isolates. Two isolates with minimal divergence (7 SNP), obtained from different samples of the same patient (PAT_72) hospitalised at 2SUR and ORTHO−29C. These isolates displayed an identical resistance gene profile characterised by the presence of *bla*_OXA-2_, consistent with within−host microevolution. The remaining two isolates formed a genetically diverse sublineage, each separated from the other ST235 isolates by several hundred SNP with broader variability in resistance determinants, including unique combinations of *bla*_OXA−17_, *bla*_OXA−210_, together with additional aminoglycoside and fluoroquinolone resistance determinants that varied between the two isolates.

Isolate 8197B_2024 was highly divergent, showing SNP distances ranging from 628 to 755 SNP when compared with the six isolates in the first phylogenetic branch and from 722 to 2949 SNP when compared with the four isolates forming the second branch. Its resistome included *bla*_VIM−1_, *bla*_OXA−488_, *bla*_PDC−374_, *aac(6’)−Ib7*, together with other additional resistance determinants.

Across all ST235 isolates, the virulence gene profile was highly conserved. Every isolate carried *exoT*, *exoU*, and all except one possessed *exoY*. None of the isolates encoded *exoS*.

#### 3.3.4 Comparative phenotypic and genomic overview of major sequence types

Across lineages, XDR profiles were the most frequent in ST233 (25/25) and ST357 carrying *bla*_VIM−2_ (5/5). In ST357 *bla*_IMP−7_-positive sublineage, 60% (26/43) of isolates were XDR. This was the only sublineage in which pan-resistance occurred (2/43).

**Figure 5:**
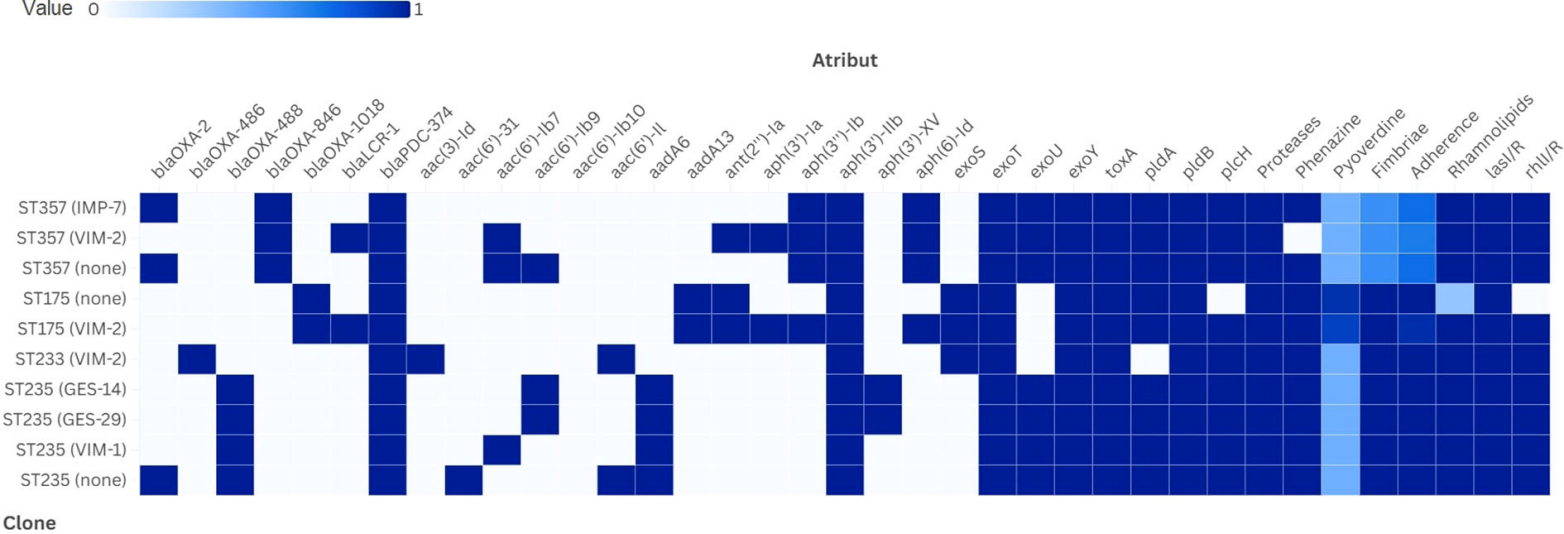
Association of important genetic determinants in PSAE sublineages. Sublineages are labelled by sequence type and the detected carbapenemase variant; “none” indicates that no carbapenemase was detected in that sublineage. For individual genes, blue squares indicate gene presence in all isolates of the corresponding sublineage, while white squares indicate absence. Colour intensity represents the proportion of genes detected in each sublineage relative to the total number of detected genes included in each functional category in this analysis. Functional categories include proteases (*aprA*, *lasA*, *lasB*), phenazine (*pvcA*–*D*, *ptxR*), pyoverdine (*fpv* and *pvd* genes), fimbriae (*fim* genes), and adherence/pili (*pil* genes).

The highest counts of virulence−associated genes were observed in ST175 (n = 273–281). Genomic characteristics of the ST sublineages are summarised in **Figure 5**.

#### 3.3.5 Genetic background of *bla*_GES_, *bla*_IMP_ and *bla*_VIM_ from short read data

Interpretation of the genetic background of class A and B beta-lactamase genes was limited by the fragmented nature of short−read assemblies. For consistency, CARD/ResFinder nomenclature is used throughout the manuscript; alternative gene names detected by BLAST (e.g. *aacA4* for *aac(6’)−Ib*; *aph*(3*’’)−Ib* and *aph(6)−Id* for *strA* and *strB*) are mentioned only when describing integron structure.

In ST357 isolates, *bla*_IMP−7_ was located within an In−p110–like class 1 integron previously associated with a Tn*4380*−related element on the LESGI−3 genomic island (Papagiannitsis et al., 2017). The conserved core (*intI1*–*aacA4*– *bla*_IMP−7_) was recovered in isolates with longer contigs, whereas shorter assemblies contained only partial *aacA4* and *bla*_IMP−7_, preventing resolution of the full cassette array and flanking regions. These truncated assemblies remain compatible with an In−p110−type structure, but the complete integron structure could not be reconstructed from short−read data.

The *bla_VIM_*_−2_ gene was detected in five ST357 isolates, twenty−five ST233 isolates, five ST654 isolates and one ST175 isolate. In ST357 isolates, the *bla*_VIM−2_ gene was located on short contigs that contained only the cassette and part of the 5′ conserved segment, precluding reliable assignment to a specific integron type. In ST233 isolates, *bla*_VIM−2_ was embedded in a Tn*402*−like class 1 integron with a conserved cassette array (*intI1*, *aac(6’)−II*, *bla*_VIM−2_, *dfrB5*, *aac(3)−Id*) and a downstream *tni* region consistent with a truncated Tn*402*−derived transposition module (Partridge et al., 2009; Tato et al., 2010; Fortunato et al., 2023). In ST654, *bla*_VIM−2_ was associated with class 1 integron showing similarity to the upstream region of Tn*As1*-linked structures, although downstream segments were truncated. In the single *bla*_VIM−2_−positive ST175 isolate, only minimal flanking sequence was recovered, preventing contextual interpretation.

Five isolates carried *bla*_GES−5_ and one isolate carried *bla*_GES−29_. All six isolates shared an identical genetic environment corresponding to the previously described In717, which is associated with a Tn*4380*−like transposon on the LESGI−3 genomic island (Papagiannitsis et al., 2017). Only partial integron structures were recovered, with consistent truncation of the 5′ conserved segment and incomplete downstream flanking regions.

The *bla*_VIM−1_ gene detected in a single ST235 isolate was located within a class 1 integron containing *intl1*, *aacA4* and *bla*_VIM−1_, with downstream alignment to an IS*6*−like element and a mercury−resistance operon.

#### 3.3.6 Genetic background of *bla*_GES_, *bla*_IMP_ and *bla*_VIM_ of representative isolates

**Figure 6:**
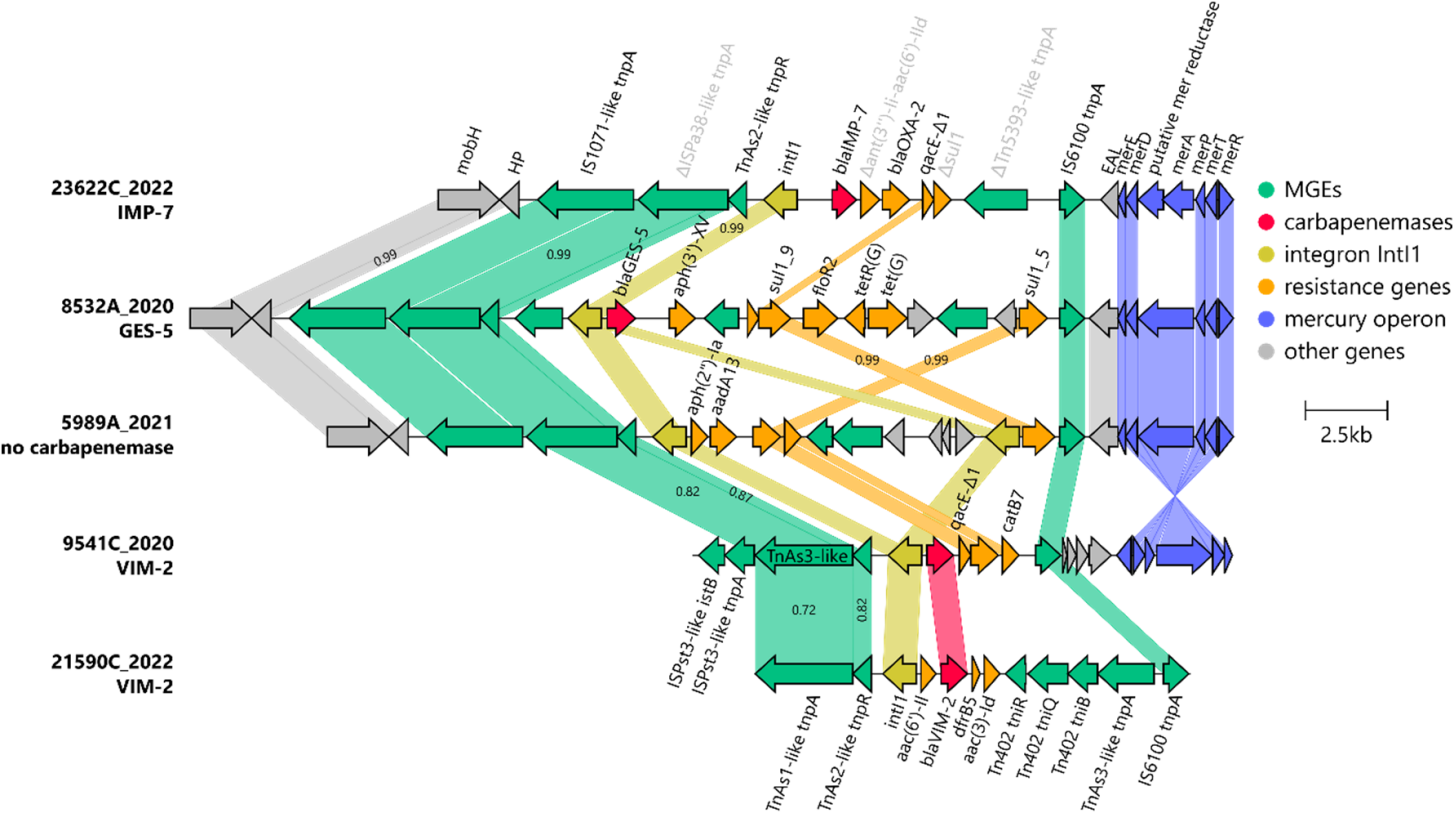
Genetic context of class 1 integron-associated resistance cassettes. Genetic surroundings of class 1 integron resistance cassette in chromosomes of five representative isolates from this study. Arrows show coding sequences (CDS) of mobile genetic elements (MGEs) (green), carbapenemases (red), other resistance-associated genes (orange), integron (yellow), mercury operon (blue) and other genes (grey). Links are drawn if the similarity of two CDS exceeds 70%, if the linked CDS are not identical, the similarity is highlighted within the link.

Four of the five selected representatives subjected to long-read sequencing carried an important carbapenemase gene (23622C_2022, 9541C_2020, 21590C_2021, 8532A_2020) and last of the isolates (5989A_2020) carried an aminoglycoside-conferring gene which was incorporated in the same region as the carbapenemases (**Figure 6**). The genes of interest were embedded within a class 1 integron in chromosomes of all five isolates. In these isolates, the class 1 integron genetic cassettes were flanked by Tn*3* family elements, and by IS*6100* element followed by mercury resistance operon, with an exception of isolate 21590C_2021 where mercury operon was not present (**Figure 6**).

The cassette associated with *bla*_IMP−7_ in isolate 23622C_2022 consisted of *intI1*, *bla*_IMP−7_, *Δant(3’’)-Ii-aac(6’)-IId*, *bla*_OXA−2_, *qacEΔ1*, and *Δsul1.* The genetic cassette linked to *bla*_GES-5_ in 8532A_2020 comprised of *intI1*, *bla*_GES−5_, and *aph(3′)-XV* interrupted by IS*Pa21*-like element which was followed by *qacEΔ1*, *sul1* (AY963803), *floR2*, *tetR*(G), and *tet*(G). Genetic context of *bla*_VIM-2_ differed between the two VIM-2-encoding isolates (9541C_2020 and 21590C_2021). The resistance cassette in isolate 9541C_2020 was formed by *intI1*, *bla*_VIM-2_, *qacEΔ1*, *sul1* (EU780013), and *catB7*, while in isolate 21590C_2021, the cassette included *intI1*, *aac(6’)-II*, *bla*_VIM-2_, *dfrB5*, and *aac(3)-Id*. Isolate 5989A_2021 carrying a class 1 integron resistance cassette without a carbapenemase was composed of *intI1*, *aph(2”)-Ia*, *aadA13*, *sul1* (EU780013), and *catB7*. This isolate carried additional *intI1* element containing *sul1* (AY963803) (**Figure 6**).

The comparison with publicly available PSAE sequences (CP056774, KY860566, KY860570 and KY860573) from the Czech Republic collected in 2015 (Papagiannitsis et al., 2017) revealed that the genetic surroundings of carbapenemase genes are highly conserved. In the analysed region, the pairwise identity of isolate 23622C_2020 carrying *bla*_IMP-7_ and KY860566 was 99.96% previously linked to In-p110. The GES-5-encoding 8532A_2020 isolate showed a similarity of 99.99% to KY860573, carrying *bla*_GES-5_ within the In717-like integron. While the pairwise identity of 9541C_2020 and KY860570 was 99.8% with several SNPs in IS*Pst3*-like element linked to the In56-like integron, the VIM-2 surroundings in the 21590C_2022 were identical with the described genetic context in CP056774 associated with truncated Tn*402*-like element, confirming the findings in fragmented short-read assemblies.

## 4 Discussion

Our dataset comprised 157 meropenem-resistant PSAE isolates, all of which met the MDR definition by Magiorakos et al (Magiorakos et al., 2012; Tamma et al., 2024). However, according to study of Kadri et al. describing the difficult-to-treat (DTR) concept (Kadri et al., 2018), 127 isolates were categorised as DTR, reflecting combined resistance to penicillins, cephalosporins, carbapenems and fluoroquinolones. When applying the PSAE−specific criteria proposed by Cosentino et al. (2023), 134 isolates fulfilled the DTR definition based on nonsusceptibility to key first−line agents. The high proportion of DTR isolates in our cohort underscores the limited therapeutic options available for PSAE in our setting. Notably, 81 of 157 isolates also met the XDR definition, further highlighting the clinical severity of resistance profiles and reinforcing the need for surveillance frameworks that extend beyond the MDR classification.

Three major high-risk lineages (ST357, ST175 and ST233) circulated concurrently across both hospitals (UHO and MHO), with the strongest overlap observed between the PA-ICU and DARIC units. This pattern likely reflects frequent patient transfers both within and across these facilities. Although ST175 was the most common lineage by isolate count, ST357 affected the highest number of patients, indicating broader dissemination despite a similar sampling period. ST233 also contributed substantially to the population structure. Together, these three lineages accounted for more than 80% of all isolates, underscoring their dominant role in the local PSAE population.

ST357 is recognised as a high−risk PSAE lineage, yet contemporary genomic data from Europe remain limited. Apart from the early reports of ST357 isolates carrying *bla*_IMP−7_ and *bla*_VIM-2_ in the Czech Republic (Hrabák et al., 2011; Papagiannitsis et al., 2017), recent European reports have focused mainly on localised outbreaks (Querin et al., 2025) rather than broader population-level genomic analysis. The isolates collected between 2020 and 2024 in this study therefore provide an overview of ST357 circulating in Central Europe.

ST357 has previously been identified as the dominant carbapenemase−producing PSAE lineage in the Czech Republic, with Papagiannitsis et al. reporting 120 isolates and demonstrating clonal spread of *bla*_IMP−7_−positive strains across multiple hospitals, alongside a small *bla*_VIM−2_ sublineage (Papagiannitsis et al., 2017). Our SNP−based analysis shows that ST357 circulating in Olomouc hospitals is more heterogeneous, forming two deeply divergent clusters defined by *bla*_VIM−2_ and *bla*_IMP−7_, separated by several thousand SNP.

The *bla*_VIM−2_−positive isolates (n=5) encoded only OXA−846 class D beta−lactamase. All *bla*_VIM−2_−positive ST357 isolates exhibited XDR phenotype, remaining susceptible only to colistin, a pattern fully consistent with their genomic background. The high similarity (99.8%) of a long-read sequenced representative isolate (9541C_2020) with a previously reported genetic context (Papagiannitsis et al., 2017) confirmed an In56 class 1 integron embedded within a Tn*3*−family transposon consistent with the PAGI−56. Loss of *oprD*, a well−established contributor to carbapenem resistance, was observed in all isolates (Amisano et al., 2025; Wang et al., 2025). All *bla*_VIM−2_ ST357 isolates also lacked *soxR*, the regulator of the *mexGHI*−*opmD* efflux operon, which is primarily associated with quorum sensing, intercellular communication and biofilm physiology rather than antibiotic resistance (Dietrich et al., 2006; Lorusso et al., 2022). The absence of *ptxR* and the *pvcA–D* paerucumarin biosynthetic cluster further indicates disruption of phenazine− and quorum−sensing–linked secondary metabolism (Carty et al., 2006; Clarke-Pearson and Brady, 2008). Together, these features represent a coordinated loss of regulatory and metabolic elements associated with biofilm maturation, consistent with studies showing that phenazine−deficient mutants form thinner and less structured biofilms (Dietrich et al., 2006; Price-Whelan et al., 2006; Ramos et al., 2010; Dietrich et al., 2013).

All *bla*_IMP−7_−positive ST357 isolates (n=43) displayed a largely conserved resistance profile, with uniform resistance to all major antipseudomonal agents and variability restricted to piperacillin–tazobactam, amikacin and colistin. Occasional pan-resistant isolates were detected, but most retained susceptibility to amikacin and/or colistin, representing the predominant phenotype of this sublineage. Following the phenotypic profile, the *bla*_IMP−7_−positive isolates consistently carried both *bla*_OXA−2_ and *bla*_OXA−846_, a pattern compatible with the cassette structure described by Papagiannitsis et al. (2017). The alignment of a long-read sequenced representative isolate (23622C_2022) revealed a high similarity (99.96%) in the genomic context with the published ST357 isolates.

The virulence profile reported for ST357 in previous study from the Czech Republic (Papagiannitsis et al., 2017) was consistent with our findings. The virulence repertoire was similarly conserved, with universal presence of *exoU*, *exoT* and only a single cluster lacking *exoY*, suggesting long−term stability of T3SS effector determinants within this lineage. ExoU is a potent PLA_2_ cytotoxin associated with rapid necrotic cell death (Sato and Frank, 2004) and has been linked to acute pneumonia and sepsis (Allewelt et al., 2000). Given its association with poor clinical outcomes, ExoU is increasingly investigated as a therapeutic target, with studies evaluating inhibitors capable of counteracting its cytotoxic activity (Foulkes et al., 2019; Foulkes et al., 2021). All ST357 isolates lacked multiple essential components of the pyoverdine system, indicating a fragmented and likely non−functional siderophore biosynthetic cluster. In addition, isolates of this ST carried fewer *pil* and *fim* genes than isolates of other STs, supporting a shift toward traits commonly observed in lineages adapted to long−term colonisation. This pattern suggests attenuation of siderophore− and pili−dependent virulence traits, features frequently observed in chronic−adapted lineages, while retaining ExoU-mediated acute cytotoxic potential (Smith et al., 2006; Harrison et al., 2017).

The high−resolution SNP analysis revealed substantial intra−host microevolution among *bla*_IMP−7_−positive isolates and multiple genomic clusters, including multi−patient clusters spanning several wards. These findings indicate that ST357 in the Czech Republic remains both widely disseminated and genomically diverse, comprising long−established IMP−7 sublineages and VIM−2 sublineages that continue to circulate regionally, consistent with the distribution reported by Papagiannitsis et al (Papagiannitsis et al., 2017).

Comparison with other European datasets underscores the regional specificity of our findings. The absence of NDM−positive ST357 in our setting, together with the presence of both VIM−2 and IMP−7 sublineages and evidence of intra−host evolution, is consistent with the circulation of established regional ST357 lineages. In Poland, carbapenem−resistant PSAE populations were dominated by ST111 and ST235 (Urbanowicz et al., 2021), while a French surgical intensive care unit reported ST357 carrying *bla*_VIM−4_ (Querin et al., 2025). In the Netherlands, ST357 has been reported exclusively in the context of an imported *bla*_NDM−1_−positive isolate originating from Kenya (Rossel et al., 2024), a variant not detected in our cohort.

Beyond Europe, recent genomic studies show that ST357 is globally distributed lineage with substantial variability in its acquired beta−lactamase repertoire. Reports from Saudi Arabia describe ST357 producing PDC−3, OXA−50, VEB-9 and LCR−1, but lacking IMP−7 or VIM−2 (Alshammari et al., 2026). In South Asia, an XDR ST357 isolate carried an unusually broad combination of beta−lactamase genes, including *bla*_PME−1_, *bla*_VEB−9_, *bla*_NDM−1_, *bla*_PDC−11_ and *bla*_OXA−846_ (Mondol et al., 2024). A global survey of 1,045 complete PSAE genomes found ST357 among the more frequently represented lineages (21 genomes, 2.01%) (Choudhury and Andam, 2026). Compared with these international datasets, Czech ST357 isolates showed a markedly more homogeneous carbapenemase profile dominated by *bla*_IMP−7_ and consistently carried *bla*_OXA−2_ in *bla*_IMP−7_-positive isolates and *bla*_OXA−846_ in all ST357 isolates, underscoring both the global adaptability of this lineage and the distinct regional patterns shaping its resistome.

The predominant ST175 lineage consisted almost entirely of isolates lacking MBL or GES genes, with only a single exception. This sequence type belongs to high−risk epidemiological clones associated with MDR phenotypes (Pérez et al., 2019; Pérez-Vázquez et al., 2020; Fernández et al., 2025). Although ST175 is generally linked to the presence of the *bla*_VIM−2_ gene, only one isolate in our dataset carried this determinant (Viedma et al., 2012; Pérez-Vázquez et al., 2020). All isolates except the *bla*_VIM−2_−positive one were susceptible to AMI and COL, and most were also susceptible to CZA and CNT. These findings correspond with the study by Papagiannitsis et al., which showed that ST175 isolates in the Czech Republic are highly disseminated, but typically lack class A or D beta−lactamase genes (Papagiannitsis et al., 2017). Similarly, Cabot et al. documented ST175 PSAE circulating in eight different Spanish hospitals and demonstrated that the ST175 resistome is shaped primarily by mutational events in *ampR*, *oprD* and *mexZ*, the latter resulting in MexXY overexpression (Cabot et al., 2016). In our dataset, all ST175 isolates carried a truncating mutation in *oprD*, consistent with the findings previously reported by Papagiannitsis et al (Papagiannitsis et al., 2017). Considering the MER resistance phenotype, the absence of carbapenemases and the presence of altered *oprD*, efflux activity likely contributes substantially to the observed resistance.

The characteristic *exoS^+^/exoT^+^/exoY^+^* profile typical for ST175 was observed, consistent with Papagiannitsis et al (Papagiannitsis et al., 2017). Among the analysed high−risk clones (ST357, ST233 and ST235), ST175 carried the highest overall number of virulence−associated genes. Genes associated with adherence- and biofilm-related functions were more frequent than acute virulence determinants. Given the higher proportion of *pvd*, *fim* and *pil* genes encoding pyoverdine and adherence structures, isolates of this ST may represent a lineage with a less extensive resistome but a comparatively higher capacity for adhesion and biofilm formation than ST357. In addition, most ST175 isolates lacked *rhlA*, *rhlB*, *rhlI* and *rhlR*, indicating disruption of the *rhl* quorum−sensing system and reduced rhamnolipid production. This loss is typically associated with diminished acute virulence and swarming motility but enhanced biofilm stability and persistence (Caiazza et al., 2005; Smith et al., 2006; Van Gennip et al., 2009; Bazire and Dufour, 2014; Ren et al., 2024; Elbehiry et al., 2026). Although ST175 lacks the *exoU* gene associated with acute cytotoxicity, its broader repertoire of adherence− and biofilm−related genes suggests a phenotype characterised by lower acute virulence but enhanced persistence−associated traits, which aligns with its epidemiological success and ability to establish long−term colonisation.

The exceptionally wide SNP range observed outside closely related isolate groups (up to 1566 SNPs) indicates long−term diversification within ST175 and supports the presence of multiple, distantly related sublineages circulating in the hospital environment over several years.

ST233 formed a genetically homogeneous lineage dominated by a single epidemiologically linked cluster, consistent with sustained intra−unit transmission rather than repeated introductions. All isolates carried *bla*_VIM−2_ and exhibited an XDR phenotype. The association of this ST with *bla*_VIM−2_ has been reported in multiple studies (Zafer et al., 2015; Taiaroa et al., 2018; Bitar et al., 2022; Doumith et al., 2022). ST233 is classified among internationally recognized high-risk clones (del Barrio-Tofiño et al., 2020; Flores-Vega et al., 2025), although its occurrence in Europe is largely sporadic and typically limited to isolated cases or small local clusters (Hansen et al., 2014; Papagiannitsis et al., 2017; Giani et al., 2018). The susceptibility of the isolates was restricted to colistin, reflecting a highly conserved resistance profile within this ST. The only isolate susceptible to amikacin was also the sole isolate lacking *mexX* and *mexY*, components of the MexXY−OprM efflux system, which is the principal determinant of aminoglycoside resistance in PSAE (Morita et al., 2012). The concurrence of amikacin susceptibility and absence of *mexXY* is therefore biologically plausible, yet its restriction to a single isolate suggests an isolate−specific event rather than a lineage−level characteristic. Although the remaining 24 isolates carried *mexXY* together with aminoglycoside−modifying enzymes, the presence of *mexXY* alone does not indicate efflux activity, as regulatory mutations and expression levels were not assessed. The genetic background analysis of a representative ST233 isolate (21590C_2022) revealed that *bla*_VIM−2_ is embedded within a class 1 integron linked to a Tn*402*−derived transposition module, a configuration characteristic for successful and globally disseminated VIM−2 lineages (Fortunato et al., 2023).

The virulence gene repertoire of ST233 was limited to the T3SS effectors *exoS*, *exoT* and *exoY*, accompanied by a broad set of adherence−associated determinants and additional core virulence factors. The genomic uniformity and narrow phenotypic variability observed in our cohort indicate that ST233 represents a stable, long−standing lineage circulating locally, contrasting with the more heterogeneous ST357 and mutation−driven ST175 lineages identified in this study.

The MDR phenotype, global spread and accumulation of multiple beta−lactamases have established ST235 as a high−risk clone. Moreover, this ST is associated with production of the ExoU cytotoxin, further enhancing its pathogenic potential, and concerns regarding its presence in hospital settings are therefore increasing (Aroca Molina et al., 2024). ST235 isolates from Olomouc hospitals carried the following class A or class B beta−lactamase genes: *bla*_GES-14_ (5/11), *bla*_GES-29_ (1/11) and *bla*_VIM-1_ (1/11). To our knowledge, this represents the first detection of a *bla*_VIM-1_-positive ST235 isolate in this setting and in the Czech Republic. The remaining four ST235 isolates lacked class A or class B beta−lactamases (4/11) and instead carried *bla*_OXA-17_ (1/4), *bla*_OXA-2_ (2/4) or *bla*_OXA-210_ (1/4). As documented by Treepong et al., ST235 exhibits extensive variability in both chromosomal and acquired beta−lactamase determinants, with no characteristic class A or class B signature. Their study reported a wide range of beta−lactamase genes, including *bla*_KPC-2_, *bla_GES_*_-19_, *bla*_PER-1_, *bla*_IMP-1_, *bla*_IMP-26_, *bla*_IMP-34_, *bla*_VIM-2_, *bla*_VIM-4_ (Treepong et al., 2018). Similarly, Zhao et al. associated ST235 isolates with *bla*_VIM-2_ (Zhao et al., 2023), and the Greek study detected *bla*_VIM-2_ and *bla*_VIM-4_ in four and eleven ST235 isolates, respectively (Papagiannitsis et al., 2020). Within our study, long-read sequencing of a representative ST235 isolate revealed a high similarity of the carbapenemase genetic surroundings with previously reported ST235 isolates from Brno and Ostrava conferring the presence of *bla*_GES-5_ (equivalent to *bla*_GES-14_) within a truncated integron In717 (Papagiannitsis et al., 2017).

High diversity among ST235 isolates in the study by Treepong et al. was also observed at the level of aminoglycoside resistance genes, which is in accordance with our findings (Treepong et al., 2018). Most isolates carried the *mexXY* efflux system genes, yet the relationship between *mexXY* presence and aminoglycoside phenotype was not straightforward. One isolate lacked *mexXY* and was susceptible to amikacin, which may explain its preserved susceptibility. Another aminoglycoside−resistant isolate carried *mexXY* but no genes for major aminoglycoside−modifying enzymes. In this case, efflux−mediated resistance is a plausible explanation. However, this cannot be confirmed because regulatory mutations and expression levels were not assessed. As documented in many studies, efflux activity depends on regulatory elements (e.g., *mexZ*, *armZ*, *parRS*) and permeability changes (Morita et al., 2012; Kawalek et al., 2019). Because these data were not available, the functional contribution of *mexXY* to aminoglycoside resistance in ST235 cannot be inferred and represents a key limitation of the study. These observations highlight the substantial genomic and resistance−gene variability within ST235, which likely contributes to its successful persistence and adaptation in diverse hospital environments.

The co−existence of *exoT*, *exoU* and *exoY* was confirmed, consistent with the known virulence profile of this ST (Papagiannitsis et al., 2017; Treepong et al., 2018). ST235 isolates carried a complete cytotoxic and adherence−related virulence repertoire, characteristic of its globally described hypervirulent phenotype. Moreover, in our cohort, eight of eleven ST235 isolates were classified as etiological agents rather than colonisers, in accordance with previous reports associating this lineage with acute human infections (Fischer et al., 2020).

Across the major high−risk clones, distinct genomic and clinical patterns emerged. ST175 carried the broadest virulence gene repertoire but lacked the cytotoxic *exoU* gene, whereas ST235 combined *exoU* with a higher proportion of etiological isolates, supporting its role as the most clinically aggressive lineage in our cohort. ST357 and ST175 were also frequently associated with infection rather than colonization, while ST233 was predominantly detected in colonizing isolates despite its uniform XDR phenotype. ST357 *bla*_IMP-7_ sublineage harboured two isolates exhibiting pan-resistance. We observed that the MDR PSAE population across both hospitals is shaped by a set of epidemiologically successful high−risk lineages, each defined by a characteristic resistance and virulence traits, underscoring the importance of integrated genomic and clinical surveillance. These findings indicate that assessing the clinical impact of MDR PSAE requires integrating phenotypic resistance, genomic background, and key virulence determinants rather than resistance genes alone.

Although multiple RND efflux systems were detected, their functional relevance could not be assessed because expression and regulatory data were not available. This represents a limitation of the study, as efflux−mediated resistance cannot be inferred from genomic presence alone (Lister et al., 2009; Fernández and Hancock, 2012).

## 5 Conclusion

This study provides the first updated genomic analysis of MDR PSAE in the Czech Republic since 2017, based on isolates collected between 2020 and 2024. Moreover, this study documents the first detection of a *bla*_VIM−1_-positive ST235 isolate in UHO and in the Czech Republic. Our findings highlight the dominance of high−risk clones, the persistence of ST−specific virulence signatures and the importance of integrating virulence profiling into genomic surveillance. Together, these results underscore the value of comprehensive molecular epidemiology for informing infection−control measures and supporting therapeutic decision−making in settings with a high burden of MDR PSAE.

## Data Availability

The datasets presented in this study can be found in online repositories. All genome assemblies have been deposited in GenBank under BioProject PRJNA1443687.
