# Supplementary figures and images for: High−risk lineages shape the resistome and virulome of multidrug−resistant Pseudomonas aeruginosa
Source: Front Cell Infect Microbiol. 2026 Jun 17;16:1843668. doi: 10.3389/fcimb.2026.1843668 (PMC13319044; doi:10.3389/fcimb.2026.1843668)

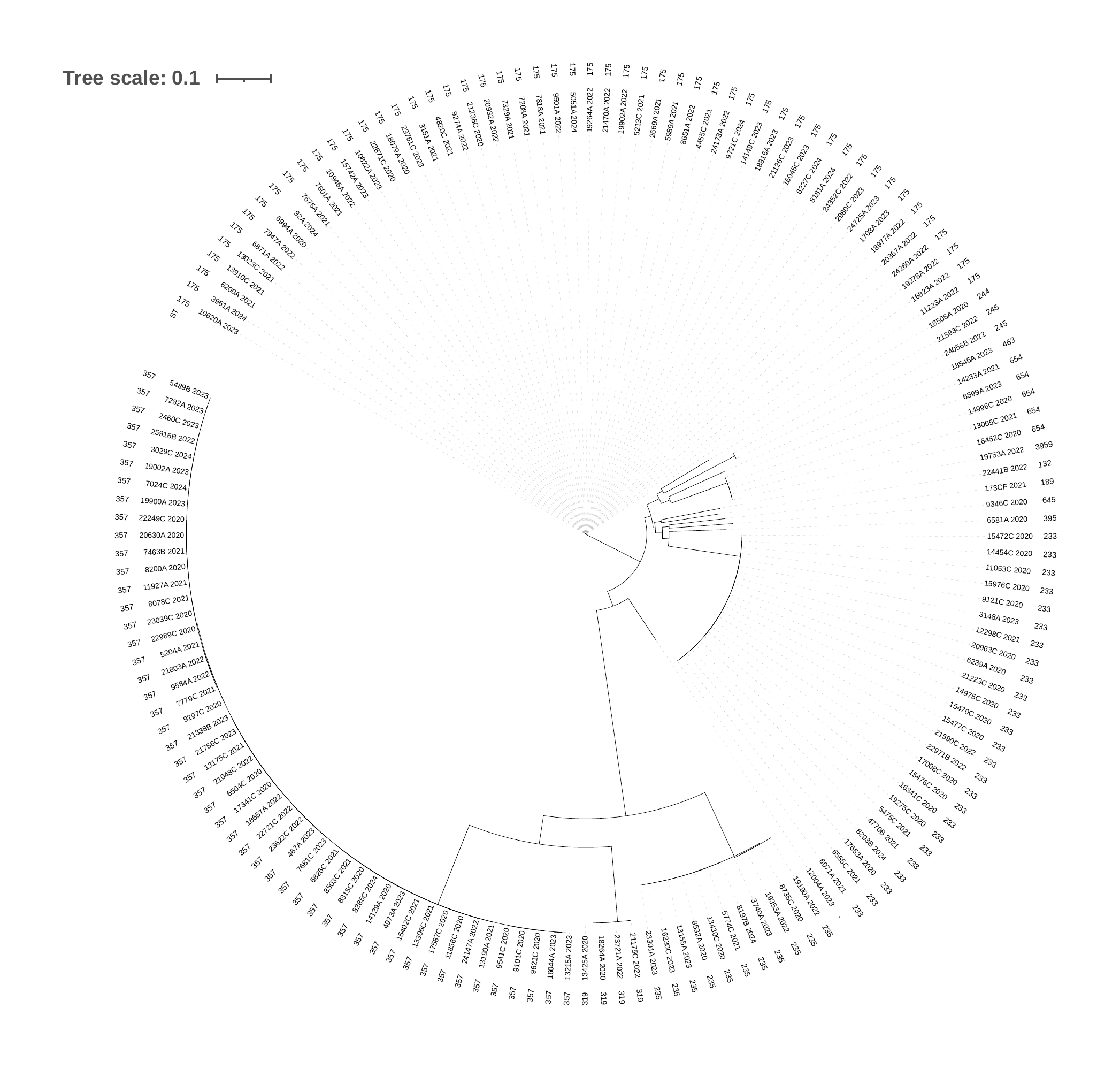

Supplement: Supplementary file 1 [file Image1.tiff]
