# Supplementary material for: High−risk lineages shape the resistome and virulome of multidrug−resistant Pseudomonas aeruginosa
Source: Front Cell Infect Microbiol. 2026 Jun 17;16:1843668. doi: 10.3389/fcimb.2026.1843668 (PMC13319044; doi:10.3389/fcimb.2026.1843668)
Supplement: Supplementary file 2 [file Table1.docx]

Supplementary Table S1. Types of samples.

| Type of sample | Prevalence |
| --- | --- |
| urine | 31.8% (50/157) |
| lower respiratory tract | 30.6% (48/157) |
| wound swabs | 14.6% (23/157) |
| upper respiratory tract | 8.9% (14/157) |
| cannula, drain fluid, punctate, soft tissue | 7.6% (12/157) |
| haemoculture | 4.5% (7/157) |
| stool | 1.9% (3/157) |

Supplementary Table S2. Department abbreviations used in this study.

| Department abbreviation | Department |
| --- | --- |
| 1IM-4 | 1st Department of Internal Medicine – 4th Ward |
| 1IM‑ICU | 1st Department of Internal Medicine – Intensive Care Unit |
| 1SUR-8 | 1st Department of Surgery – 8th Ward |
| 1SUR‑9 | 1st Department of Surgery – 9th Ward |
| 1SUR‑OPD | 1st Department of Surgery – Outpatient Department |
| 2IM-30 | 2nd Department of Internal Medicine – 30th Ward |
| 2IM-ICU | 2nd Department of Internal Medicine – Intensive Care Unit |
| 2SUR | 2nd Department of Surgery |
| 2SUR‑VASC | 2nd Department of Surgery – Vascular Outpatient Department |
| 3IM‑39A | 3rd Department of Internal Medicine – 39A Ward |
| 3IM‑39C | 3rd Department of Internal Medicine – 39C Ward |
| 3IM-ICU | 3rd Department of Internal Medicine – Intensive Care Unit |
| 3IM-TRANSP | 3rd Department of Internal Medicine – Transplantation Outpatient Department |
| DARIC PA-ICU | Department of Anaesthesiology, Resuscitation and Intensive Care Medicine – Post‑acute Intensive Care Unit |
| DARIC‑15 | Department of Anaesthesiology, Resuscitation and Intensive Care Medicine – 15th Ward |
| DARIC-SDU | Department of Anaesthesiology, Resuscitation and Intensive Care Medicine – Postoperative Step‑Down Unit |
| DARIC-SICU | Department of Anaesthesiology, Resuscitation and Intensive Care Medicine – Surgical Intensive Care Unit |
| ED‑SOU | Emergency Department, Surgical Observation Unit |
| HEMONC‑5A | Department of Haemato‑Oncology – 5A Ward |
| ICU | Intensive Care Unit |
| IM‑ICU | Internal Medicine – Intensive Care Unit |
| LTCF | Long‑Term Care Facility |
| LTCU | Long‑term Care Unit |
| LT‑ICU | Long‑term Intensive Care Unit |
| NEUR-35 | Department of Neurology – 35th Ward |
| NSG-31B | Department of Neurosurgery – 31B Ward |
| NSG‑ICU | Department of Neurosurgery – Intensive Care Unit |
| ONCO-42A | Oncology clinic – 42A Ward |
| ORTHO-29C | Department of Orthopaedics – 29C Ward |
| PA-ICU-A | Post‑acute Intensive Care Unit – A Ward |
| PED‑21A | Paediatrics Clinic – 21A Ward |
| PULM-25 | Department of Pulmonary Diseases and Tuberculosis – 25 Ward |
| PULM-26 | Department of Pulmonary Diseases and Tuberculosis – 26 Ward |
| PULM-ICU | Department of Pulmonary Diseases and Tuberculosis – Intensive Care Unit |
| PULM-OPD | Department of Pulmonary Diseases and Tuberculosis – Outpatient Department |
| TRAUMA-27 | Department of Traumatology – 27th Ward |
| UROL-20 | Department of Urology – 20th Ward |
| UROL‑ADM | Department of Urology – Admission Unit |
| UROL-ICU | Department of Urology – Intensive Care Unit |
| UROL-OPD | Department of Urology – Outpatient Department |

Supplementary Table S3. Prevalence of all detected genes conferring resistance to beta-lactams and aminoglycosides

| Class of enzyme | Gene variant | Prevalence |
| --- | --- | --- |
| class A beta-lactamase | *bla*_GES-14_ | 3.2% (5/157) |
|  | *bla*_GES-29_ | 0.6% (1/157) |
| class B beta-lactamase | *bla*_IMP-7_ | 27.4% (43/157) |
|  | *bla*_VIM-2_ | 22.9% (36/157) |
|  | *bla*_VIM-1_ | 0.6% (1/157) |
| class C beta-lactamase | *bla*_PDC-374_ | 93.6% (147/157) |
|  | *bla*_PDC-46_ | 1.9% (3/157) |
|  | *bla*_PDC-55_ | 3.8% (6/157) |
|  | *bla*_PDC-590_ | 0.6% (1/157) |
|  | *bla*_LCR-1_ | 3.8% (6/157) |
| class D beta-lactamase | *bla*_OXA-2_ | 29.3% (46/157) |
|  | *bla*_OXA-17_ | 0.6% (1/157) |
|  | *bla*_OXA-210_ | 0.6% (1/157) |
|  | *bla*_OXA-486_ | 15.9% (25/157) |
|  | *bla*_OXA-488_ | 7.0% (11/157) |
|  | *bla*_OXA-1018_ | 33.8% (53/157) |
|  | *bla*_OXA-846_ | 31.2% (49/157) |
|  | *bla*_OXA-396_ | 3.2% (5/157) |
|  | *bla*_OXA-494_ | 1.9% (3/157) |
|  | *bla*_OXA-847_ | 0.6% (1/157) |
|  | *bla*_OXA-901_ | 2.5% (4/157) |
|  | *bla*_OXA-905_ | 0.6% (1/157) |
|  | *bla*_OXA-1027_ | 0.6% (1/157) |
|  | *bla*_OXA-1127_ | 0.6% (1/157) |
|  | *bla*_OXA-1244_ | 0.6% (1/157) |
|  | *bla*_OXA-1283_ | 0.6% (1/157) |
|  | *bla*_OXA-1292_ | 0.6% (1/157) |
| N-acetyltransferase | *aac(3)-Id* | 15.9% (25/157) |
|  | *aac(6')-31* | 1.9% (3/157) |
|  | *aac(6')-Ib10* | 6.4% (10/157) |
|  | *aac(6')-Ib7* | 7.0% (11/157) |
|  | *aac(6')-Ib9* | 5.7% (9/157) |
|  | *aac(6')-Il* | 18.5% (29/157) |
| nucleotidyltransferase | *ant(2'')-Ia* | 38.2% (60/157) |
| adenylyltransferase | *aadA13* | 31.20% (49/157) |
|  | *aadA6* | 6.40% (10/157) |
| O‑phosphotransferase | *aph(3')-VIa* | 1.3% (2/157) |
|  | *aph(3'')-Ib* | 35.70% (56/157) |
|  | *aph(3')-IIb* | 100.00% (157/157) |
|  | *aph(3')-Ia* | 3.80% (6/157) |
|  | *aph(3')-XV* | 3.80% (6/157) |
|  | *aph(6)-Id* | 35.70% (56/157) |

Supplementary Table S4 Coverage and identity of the *oprD* gene among isolates belonging to the most prevalent STs.

| ST | Carbapenemase genes | Phenotypic resistance | *oprD* % coverage (C)  and identity (I) |
| --- | --- | --- | --- |
| 357 | *bla*_IMP-7_ (43/49) | PPT (26/43), CTZ, CPM, MER, TOB, AMI (15/43), COL (3/43), CIP, CZA, CNT | C (99.55), I (90.02 – 92.34) |
|  | *bla*_VIM-2_ (5/49) | PPT, CTZ, CPM, MER, AMI, TOB, CIP, CZA, CNT | oprD not detected |
|  | no gene detected (1/49) | PPT, CTZ, CPM, MER, TOB, CIP | C (99.55), I (90.02) |
| 175 | no gene detected (52/53) | PPT, CTZ (51/52), CPM (49/52), MER, TOB (51/52), CIP, CZA (8/52), CNT (6/52) | C (99.55), I (92.34) |
|  | *bla*_VIM-2_ (1/53) | PPT, CTZ, CPM, MER, AMI, TOB, CIP, CZA, CNT | C (99.55), I (92.34) |
| 233 | *bla*_VIM-2_ (25/25) | PPT, CTZ, CPM, MER, TOB, AMI (24/25), CIP, CZA, CNT | C (99.40), I (92.26 – 92.33) |
| 235 | *bla*_GES-14_ (5/11) | PPT, CTZ, CPM (3/5), MER, TOB, AMI (3/5), CIP, CZA (2/5), CNT | C (99.92), I (95.80) |
|  | *bla*_GES-29_ (1/11) | CTZ, CPM, MER, TOB, AMI, CIP, CNT | C (99.92), I (95.80) |
|  | *bla*_VIM-1_ (1/11) | PPT, CTZ, CPM, MER, TOB, CZA, CNT | C (100.00), I (95.87) |
|  | no gene detected (4/11) | PPT, CTZ (3/4), CPM, MER, TOB, AMI, CIP, CZA (1/4), CNT (2/4) | C (99.92 – 100.00),  I (95.37 – 95.80) |
